# Report of a successful ongoing pregnancy as a result of IMSI with assisted oocyte activation

**DOI:** 10.1186/s12978-015-0031-x

**Published:** 2015-05-03

**Authors:** Bogdan Doroftei, Mihaela Zlei, Gabriela Simionescu, Radu Maftei, Simona Cumpata, Geraldine Emerson

**Affiliations:** Obstetrics and Gynecology Department, University of Medicine and Pharmacy, Iasi, Romania; Teaching Hospital Obgyn “Cuza Voda”, Iasi, Romania; Origyn Fertility Center, Iasi, Romania; Regional Institute of Oncology, Iasi, Romania; Laboratory of Molecular Biology, Regional Institute of Oncology, Romania, 2-4 Berthelot Street, postal code 700483 Iasi, Romania

**Keywords:** Globozoospermia, Artificial Oocyte Activation, IMSI

## Abstract

We report a successful ongoing pregnancy obtained in a case of total globozoospermia after intracytoplasmic morphologically selected sperm injection (IMSI) with oocyte activation. The first semen analysis on investigation showed partial globozoospermia. However, under high magnification assessment at oocyte retrieval only round headed sperm were observed. Considering the high risk of a complete failure to fertilize from IMSI the couple gave written informed consent to the use of oocyte activation media post IMSI. One embryo fertilized, developed to a hatching blastocyst and was transferred resulting in an ongoing pregnancy. This successful outcome shows the use of IMSI is useful in the evaluation of total globozooozpermia and therefore aids in the justification of the use of oocyte activation media.

**Electronic supplementary material:**

The online version of this article (doi:10.1186/s12978-015-0031-x) contains supplementary material, which is available to authorized users.

## Background

In globozoospermia the main morphological defect is characterized by the absent or severely malformed acrosome. The pathogenesis occurs during spermiogenesis and probably originates in the acrosomic vesicle fusion impairment and cytoskeleton disorders, although precise mechanisms remain to be determined [1]. Total (100% round headed spermatozoa) or partial (less than 100%) globozoospermia have been described [2,3].

The introduction of intracytoplasmic sperm injection (ICSI) and then morphologically selected sperm injection (IMSI) lead the way for males with severe globozoospermia to have the ability to father their own children. However, rates of fertilization remained poor for this cohort of males and it quickly became evident that round headed sperm did not have the ability to trigger oocyte activation [4-6]. In 1997, the first reports of assisted oocyte activation (AOA) and improved fertilization could be achieved by applying calcium ionophore in such cases [7]. Here we will report on a successful ongoing pregnancy after IMSI with oocyte activation.

## Case presentation

**Figure 1:**
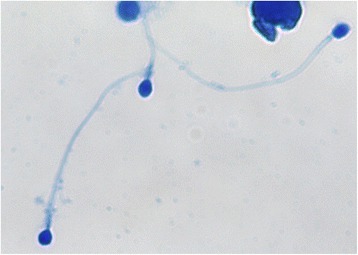
Microscopy image of sperm morphologic assessment.The staining was performed with SpermBlue (Microptic, Spain). Magification with the oil immersion, 100 x objective (Leica GX, L3200, Leica Application Capture Software).

A 32 years old couple attended for fertility consultation reporting a history of 5 years trying for pregnancy with no success. On investigation of the female the fertility workup included: check ovarian reserve by anti-mullerian hormone 3.72 ng/mL and antral follicle count 18 follicles on both ovaries, ultrasound examination of uterus and office diagnostic hysteroscopy. All showed no abnormalities. Semen analysis on the male showed a volume of 5.6 mL, concentration of 23 million/mL, 44% motile of which only 5% were progressively motile. Sperm morphology was reported as 2% normal with evidence of acrosome abnormalities (strict criteria, Figure 1). DNA fragmentation index was 10.6% and high DNA stainability was 14.9%. Both were found by flow-cytometry method.

Male factor infertility was diagnosed and the couple counseled for IMSI. Informed consent was obtained. No history was known regarding other male family members fertility.

The female patient was treated with antagonist protocol using ganirelix (Orgalutran – MSD) and the administration of 150 IU rFSH (Puregon - MSD) daily for 9 days. Oocyte retrieval was undertaken 36 hours post hCG priming 10,000 IU (Pregnyl - MSD). A total of 7 oocytes were collected of which 5 were mature and suitable for injection. Semen analysis on the day of oocyte retrieval on high magnification using an inverted microscope equipped with Nomarski differential interference contrast optics (Leica AM 6000) showed no normal morphology only total round heads (globozoospermia). Due to the concern of a total failure to fertilize the couple were informed that IMSI would be the method of choice to continue to observe the sperm sample, trying to identify any sperm with a partial or small acrosome to avoid the use of oocyte activation media. However none were observed and post IMSI the use of oocyte activation media was undertaken. The couple were fully informed of the limited data available on the use of calcium ionophore with regard to the long-term health of resultant offspring. Written consent was obtained to carry out this procedure. The characteristics of the sperm used for insemination showed total round head nucleus with no acrosome present, however all were motile.

**Figure 2:**
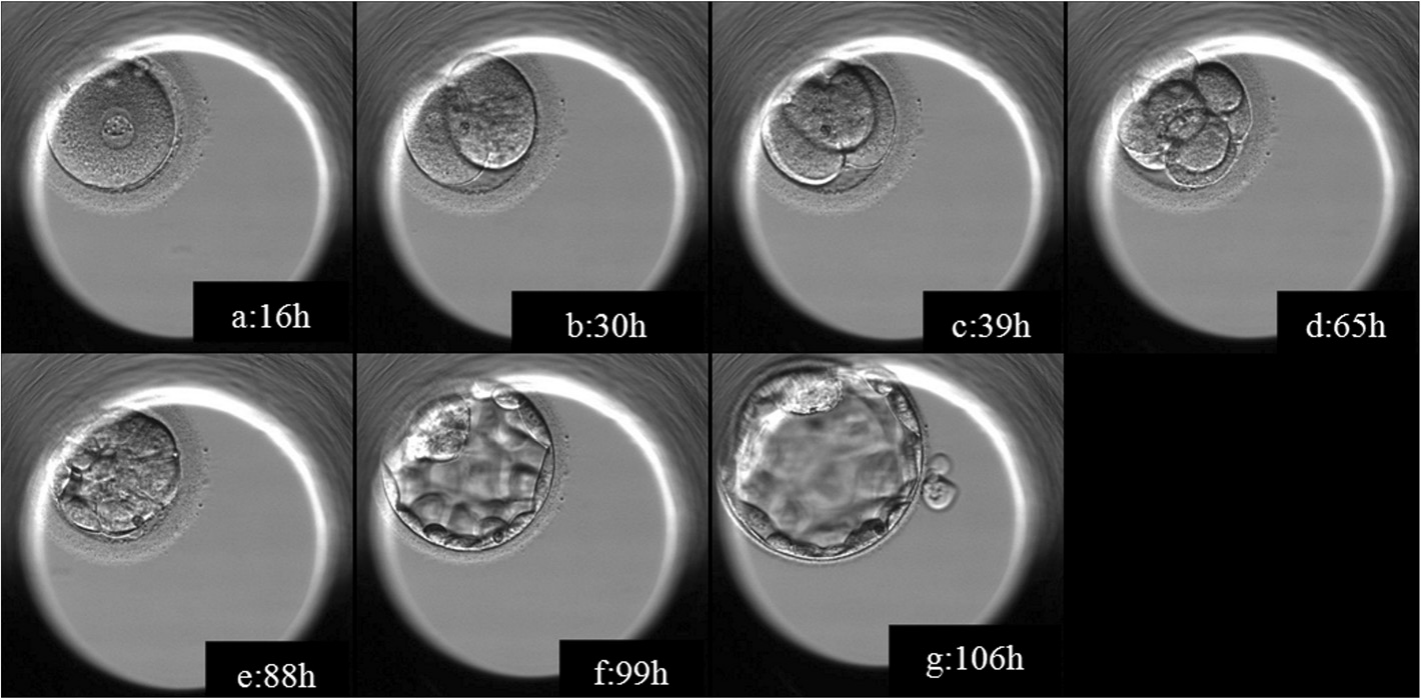
Different stages of embryo development captured with a Time Lapse System. The images were captured (Embrioscope, Fertilitech-Unisence Denmark, Embryo-Viewer Software) at different stages of the embryo’s development post injection: **a** – 2 pro-nucleai, 16 hours, **b** – 2 cells (30 hours), **c** – 4 cells (39 hours), **d** – 8 cells (65 hours), **e** – start of blastocyst (88 hours), **f** – expanded blastocyst (99 hour), **g** – hatching blastocyst (106 hours).

Following IMSI the 5 oocytes were placed into oocyte activation media (GM508, Cult Active, GYNEMED) for 15 minutes at 37 degrees 5% 02 and 6% CO2. The oocytes were then washed free of calcium ionophore through 8 drops of culture media. The oocytes were cultured over night in CSCM media (Irvine Scientific, Santa Ana, CA) in the embryoscope time lapse incubator (Fertilitech-Unisence Denmark). Sixteen hours post injection only one oocyte showed signs of fertilization and developed normally (Figure 2; an additional movie file shows this in details, see Additional file 1).

**Figure 3:**
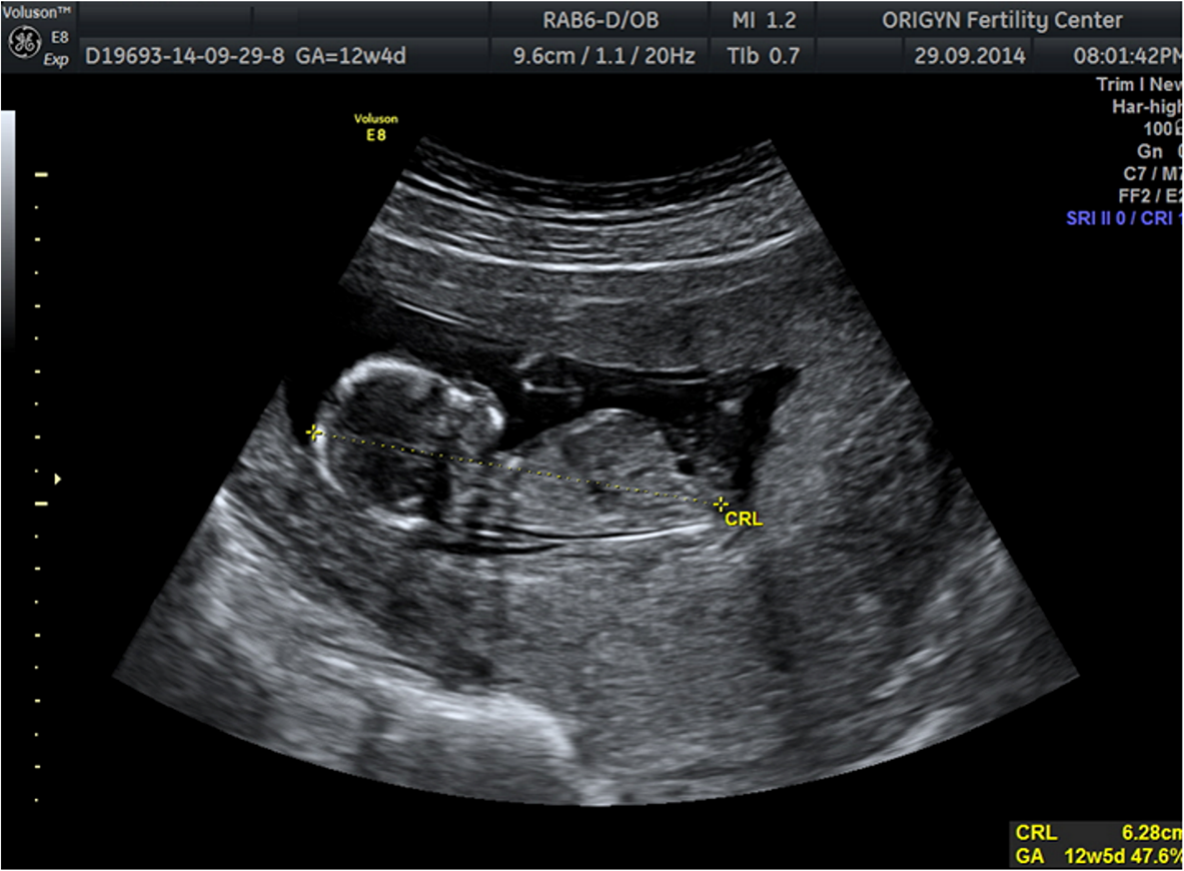
Ultrasound image of the fetus at 12 weeks and 2 days of gestation.

The embryo was cultured to day 5 uninterrupted in the embryoscope (Unisense FertiliTech, Aarhus, Danemarca) and a blastocyst grade 6AA [8] was transferred under ultrasound guidance. BhCG level on day 12 post embryo transfer was 468.9 mUI/mL, 2 days later BhCG level was 916.1 mUI/mL. Ultrasound evaluation at 12 weeks and 5 days of gestation (Figure 3) showed Fetal Heart Beat 149 bpm, CRL (crown–rump length 63 mm, nuchal transluency – 1.6 mm and intracranianal transluency 2.1 mm, ductus venosus dopller without notch, nasal bone present and tricuspid doppler in normal range. First trimester biochemical and ultrasound screening reveal low risk for trisomy.

As yet, it is unclear whether patients whose ejaculate contains both 100% globozoospermicornormal and globozoospermic cells (partial globozoospermia) suffer from a variation of the same syndrome [1] and still remains to be elucidated. Yoon et al. [9] reported absence of PLCz in both the normal and round-headed sperm of a partial globozoospermic patient, in line with the inability of partial globozoospermic sperm cell types to activate mouse oocytes as reported by Heindryckx et al. [10]. Numerous reports studying familial cases have suggested that globozoospermia is a genetic syndrome [11-15]. However, the specific mode of inheritance remains unclear, although recently it was reported that a mutation in the SPATA16 gene appears to be associated with certain types of globozoospermia in men [1].

## Conclusions

To our knowledge this is the first reported ongoing pregnancy after IMSI using globozoospermic spermatozoa with AOA in Romania. This report shows the use of high magnification tools such as IMSI in the assessment of sperm morphology can aid in the reduction of complete failure to fertilize in cases of suspected globozoospermia with the introduction of AOA when necessary.

## Consent

Written informed consent was obtained from the patient for publication of this Case report and any accompanying images. A copy of the written consent is available for review by the Editor-in-Chief of this journal.

## Additional file

Additional file 1: **Morphokinetic illustration of the embryo development, captured with a Time Lapse System.** The images were captured using the Embrioscope from Fertilitech-Unisence Denmark; the software used was Embryo-Viewer Software. Images were capture every 15 minutes, using 4 focal planes.

## Abbreviations

ICSI: Intracytoplasmic sperm injection
IMSI: Morphologically selected sperm injection
AOA: Assisted oocyte activation
